# Synergistic effects of crop residue and microbial inoculant on soil properties and soil disease resistance in a Chinese Mollisol

**DOI:** 10.1038/s41598-021-03799-3

**Published:** 2021-12-20

**Authors:** Haolang Liu, Yuqi Qi, Jihong Wang, Yan Jiang, Mingxin Geng

**Affiliations:** 1 Key Laboratory of Straw Biology and Utilization, Ministry of Education, Jilin Agricultural University, Changchun, Jilin China; 2 Institute for Environment and Climate Research, Jinan University, Guangzhou, Guangdong China

**Keywords:** Ecology, Environmental sciences

## Abstract

The soil-borne disease caused by *Fusarium graminearum* seriously affects the corn quality. Straw can greatly improve soil quality, but the effect is limited by its nature and environmental factors. This study explored the impact of straw-JF-1(biocontrol bacteria) combination on soil environment and soil disease resistance. The results showed that the combined treatment increased the proportion of soil large and small macro-aggregates by 22.50 and 3.84%, with soil organic carbon (SOC) content by 16.18 and 16.95%, respectively. Compared to treatment with returning straw to the field alone, the straw-JF-1 combination increased the soil content of humic acid, fulvic acid, and humin by 14.06, 5.50, and 4.37%, respectively. Moreover, A metagenomics showed that returning straw to the field alone increased the abundance of disease-causing fungi (*Fusarium* and *Plectosphaerella*), however, the straw-JF-1 combination significantly suppressed this phenomenon as well as improved the abundance of probiotic microorganisms such as *Sphingomonas*, *Mortierella*, *Bacillus*, and *Pseudomonas*. Functional analysis indicated that the combination of straw and JF-1 improved some bacterial functions, including inorganic ion transport and metabolism, post-translational modification/protein turnover/chaperones and function unknown, fungal functions associated with plant and animal pathogens were effectively inhibited. Pot experiments showed that the straw-JF-1 combination effectively inhibited the *Fusarium graminearum* induced damage to maize seedlings. Therefore, the combination of straw and JF-1 could be a practical method for soil management.

## Introduction

Black soils (mollisol), often referred to as the "pandas of arable land" in China, have a thick humus layer and high fertility^1^. China's black soils are one of the world's three prime maize-producing areas for their quality^2^. However, the increased intensification of farming has deteriorated the soil organic matter (SOM) content and soil quality, while the incidence of soil-borne diseases of maize is increasing. This harms food security and sustainable agricultural development^3,4^. Soil infestation of plant pathogens is often considered an important factor for the development of soil-borne diseases^5^. Stem rot, caused by *Fusarium graminearum*, is a major soil-borne disease that threatens the safe production of maize with symptoms such as stunted plant growth, root discoloration, decomposition at the seedling stage, basal rot, premature aging, and maize lodging at the adult stage^6,7^. Biological control of maize stalk rot is an environmentally friendly approach that has good potential. A variety of biocontrol bacteria and fungi have been found effective in suppressing soil-borne diseases of plants, such as *Bacillus amyloliquefaciens*, *Pseudomonas flourenscens*, and *Trichoderma gamsii*^8^.

Also, improving soil structure and quality through organic waste is an effective way to suppress soil-borne diseases from pathogenic fungi such as *Fusarium*, *Pythium*, and *Sclerotium*^9,10^. Conventionally, China is a large agricultural country that produces ~ 1.04 billion tons of straw from crop sources each year^11^. Studies showed that returning straw to the field can improve soil fertility, the composition of soil aggregates, and soil humus content^12–14^. The retention and fixation of organic matter by soil aggregates is an important driver of soil disease resistance^15^. Humus is a microbial action-derived stable form of organic matter that positively affects the microbial and crop growth, formation and stabilization of aggregates, and suppression of soil-borne diseases^16^.

Soil microorganisms play an important role in soil biological functions including biogeochemical and material cycling, and degradation and transformation of organic matter^17,18^. Some probiotic microorganisms can also promote plant growth and suppress soil-borne diseases^19,20^. Notably, straw contains some readily decomposable compounds (sugars, proteins, etc.) that can rapidly stimulate the growth of soil bacteria^21^. However, the returning straw to the field is also suggested to increase the number of pathogenic fungi (such as *Fusarium graminearum* and *Fusarium moniliforme*) and the risk of maize disease^22^. Therefore, knowledge about the response of soil microbial communities to different straw utilization practices can improve and optimize the sustainable straw-fallowing cropping patterns.

Previous studies on straw return mainly focused on its effects on soil quality and crop yield, but less on soil microbial communities, especially related to soil health. Moreover, field trials on the improved use of straw in the field, such as the addition of functional microorganisms are still lacking. In this study, straw was tested on black soils in maize growing areas with the addition of *Bacillus amyloliquefaciens* JF-1, a lab-preserved biocontrol strain with good cellulose degradation ability that efficiently inhibits *Fusarium graminearum*. In this study, we had combined application of straw and JF-1 for straw return experiment, and the major questions had explored the following: (1) examine the impact of combined application of straw and JF-1 on soil fertility, soil aggregates and soil humus; (2) investigate the effect of straw and JF-1 on soil bacterial and fungal community structure, core species and functions; (3) verify the inhibitory effect of straw and JF-1 on *Fusarium graminearum*.

## Results

### Effect of different treatments on soil nutrients

The SOC content showed a significant variation among different treatments (P < 0.05) in the following order: T2 > T3 > T1 > CK. Compared to the CK treatment, the T3 treatment significantly increased the levels of CEC, AN, AP and AK in the soil to 13.86, 11.00, 10.64 and 6.03%, respectively, the T2 treatment showed a significant increase of 4.39, 6.86, 18.02, and 7.25%, compared to the T3 treatment (P < 0.05) (Table 1). Also, the soil pH value was significantly higher in the T2 treatment than the other treatments (P < 0.05); the T3 treatment showed a significant increase compared to the CK treatment but lower than the T2 treatment (P < 0.05), the T1 and CK treatments did not show a significant difference.

**Table 1:** Effects of different treatments on soil physicochemical properties.

Treatments	SOC (g·kg^−1^)	pH	CEC (cmol kg^−1^)	AN (mg·kg^−1^)	AP (mg·kg^−1^)	AK (mg·kg^−1^)
CK	11.98 ± 0.61 c	6.07 ± 0.02 c	20.99 ± 0.26 d	82.72 ± 1.76 d	32.05 ± 1.37 c	143.36 ± 1.58 d
T1	14.26 ± 0.64 b	6.11 ± 0.01 c	22.90 ± 0.15 c	86.68 ± 2.14 c	33.53 ± 0.20 c	146.12 ± 0.60 c
T2	16.33 ± 0.45 a	6.35 ± 0.03 a	24.95 ± 0.32 a	98.12 ± 1.46 a	41.85 ± 0.90 a	163.02 ± 1.58 a
T3	16.10 ± 0.64 a	6.19 ± 0.03 b	23.90 ± 0.10 b	91.82 ± 1.07 b	35.46 ± 0.20 b	152.00 ± 1.03 b

Different letters indicate significant differences at p < 0.05 (according to Duncan’s multiple range test).

*SOC* soil organic carbon, *CEC* cation exchange capacity, *AN* available nitrogen, *AP* available phosphorus, *AK* available potassium.

### Effect of different treatments on soil agglomerate composition and carbon content

**Figure 1:**
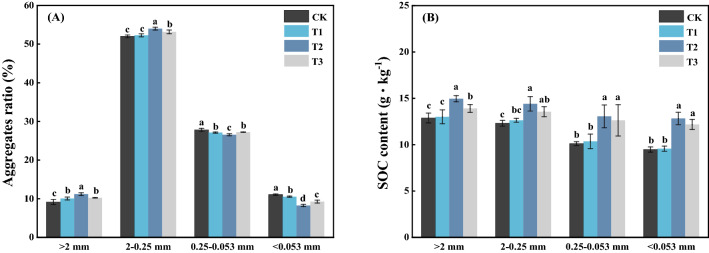
Effect of different treatments on soil agglomerate composition **(A)** and SOC content **(B)**. Different letters indicate significant differences at P < 0.05 (according to Duncan’s multiple range test).

The effects of straw and JF-1 on soil aggregates are shown in Fig. 1A. Compared with the CK treatment, the T2 treatment significantly increased the proportion of large macro-aggregates (> 2 mm; 22.50%) and small macro-aggregates (2–0.25 mm; 3.84%) but significantly decreased the proportion of micro-aggregates (0.25–0.053 mm; 4.47%) and silt/clay fraction (< 0.053 mm; 25.40%) (P < 0.05), while the T1 and T3 treatments showed no significant effect. The changes in SOC content of soil aggregates are shown in Fig. 1B. The SOC content of respective agglomerates was in the following order: large macro-aggregates (> 2 mm) > small macro-aggregates (2–0.25 mm) > micro-aggregates (0.25–0.053 mm) > silt/clay fraction (< 0.053 mm). Compared with the CK treatment, the T2 and T3 treatments significantly increased the SOC content of different sizes of aggregates by 16.18 and 8.02% (> 2 mm), 16.95 and 10.05% (2–0.25 mm), 29.14 and 24.96% (0.25–0.053 mm), and 35.38 and 28.55% (< 0.053 mm)(P < 0.05), respectively, while T1 treatment showed no effect.

### Effect of different treatments on the composition and properties of soil humic substances

**Figure 2:**
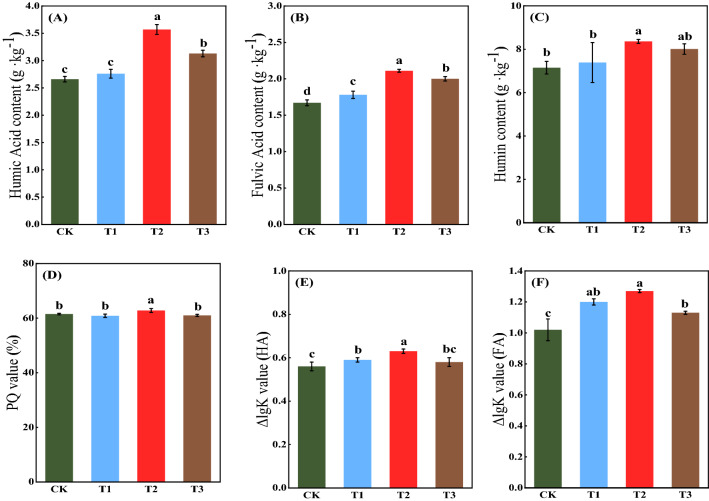
Effect of different treatments on humic acid content **(A)**, fulvic acid content **(B)**, humin content **(C)**, PQ value **(D)**, ΔlgK value (HA) **(E)** and ΔlgK value (FA) **(F)**. Different letters indicate significant differences at P < 0.05 (according to Duncan’s multiple range test).

The SOC content of the different fractions of soil humic substances in each treatment was in the following order: HM > HA > FA (Fig. 2). Compared with the CK treatment, only the T1 treatment significantly increased the SOC content of FA by 6.59% (P < 0.05). The T3 treatment significantly increased the soil carbon content of HA, FA, and HM (P < 0.05) by 17.67, 19.76, and 12.03%, respectively. Compared to the T3 treatment, the T2 treatment significantly increased the SOC content of HA, FA, and HM (P < 0.05) by 14.06, 5.50, and 4.37%, respectively. The PQ value denotes the percentage of HA in the extractable soil humic substances and reflects the degree of SOM humification. Only the T2 treatment significantly increased the PQ value compared to the CK treatment. Notably, a higher ΔlgK of humic substances suggests a simpler molecular structure of humic substances^23^. Compared with the CK treatment, the T2 treatment increased the ΔlgK of HA and FA; compared with the T3 treatment, the T2 treatment significantly increased the ΔlgK of HA and FA by 8.62 and 12.39%, respectively (P < 0.05).

### Effect of different treatments on the composition and structure of the microbial communities

**Figure 3:**
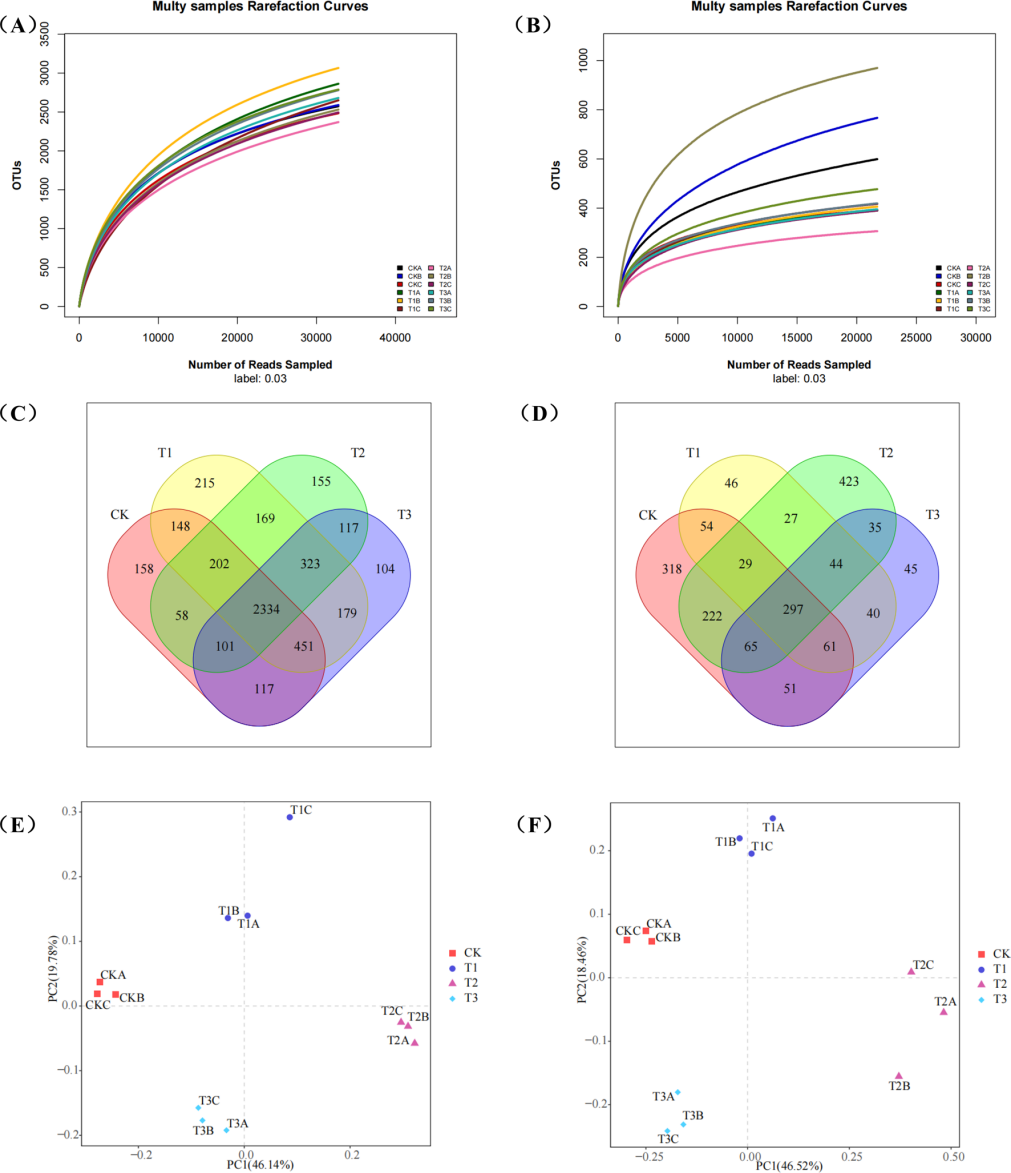
Rarefaction curves for bacteria **(A)** and fungi **(B)**. Venn diagrams of microbial communities based on OTU for bacteria **(C)** and fungi **(D)**. Bray–Curtis PCoA for bacteria **(E)** and fungi **(F)** of different treatments on OTUs level.

A total of 747,693 high-quality bacterial 16S V3–V4 sequences and 634,335 high-quality fungal ITS sequences were found in 12 soil samples. Figure 3A,B show the corresponding rarefaction curves of the bacterial and fungal communities. With the increase in the number of identified sequences, the trend at the end of the rarefaction curves stabilized. This suggests that the depth of the sequencing data was optimal. The α-diversity indexes of bacteria and fungi are shown in Table 2.

**Table 2:** Alpha diversity indexes of bacterial and fungal community in different treatments.

Treatments	Bacterial community		Fungal community	
	Chao1	Shannon	Chao1	Shannon
CK	3161.29 ± 108.58 c	9.06 ± 0.11 a	775.98 ± 286.31 a	6.26 ± 0.31 a
T1	3773.65 ± 100.68 a	8.30 ± 1.26 a	487.08 ± 24.65 a	5.38 ± 0.24 a
T2	3273.99 ± 51.46 c	8.67 ± 0.01 a	665.82 ± 422.06 a	5.77 ± 1.35 a
T3	3494.51 ± 107.48 b	9.15 ± 0.23 a	541.95 ± 44.48 a	5.95 ± 0.10 a

Different letters indicate significant differences at p < 0.05 (according to Duncan’s multiple range test).

The application of JF-1 or straw alone significantly increased the Chao1 index of soil bacteria (P < 0.05), but the combined application of the two did not show a significant effect. Figure 3C,D show the Venn diagrams of bacterial and fungal OTUs distribution in soil samples from different treatments. The number of bacterial and fungal OTUs ranged from 3459 to 4021, and 598 to 1142, respectively. Among the bacterial OTUs in different treatments, the application of JF-1 alone or straw alone significantly increased the number of OTUs and soils remediated with JF-1 alone had a maximum number of OTUs (4021, P < 0.05), but the combined application of straw and JF-1 did not show a significant effect, compared to original soils. As for fungal OTUs, no significant difference was observed between groups in different treatments. Notably, soils remediated with JF-1 alone had more unique bacterial OTUs (215), while soils remediated with JF-1-straw combination treatment had more unique fungal OTUs (423).

The PCoA based on the Bray–Curtis method showed significant differences in microbial composition at the OTU level between different treatments (Fig. 3E,F). For bacteria, two principal coordinates explained 65.92% of the variation, of which 46.14 and 10.78% belonged to PC1 and PC2, respectively. Likewise, for fungi, the two principal coordinates explained 64.98% of the variation, of which 46.52 and 18.46% belonged to PC1 and PC2, respectively. The data point distances between the JF-1 alone, straw alone, and those of the original treatment indicate some differences in the microbiota. The data points of the JF-1-straw combination showed significant deviation from the original treatment, indicating a substantial transformation of the original soil microbiota (PERMANOVA, P < 0.001).

**Figure 4:**
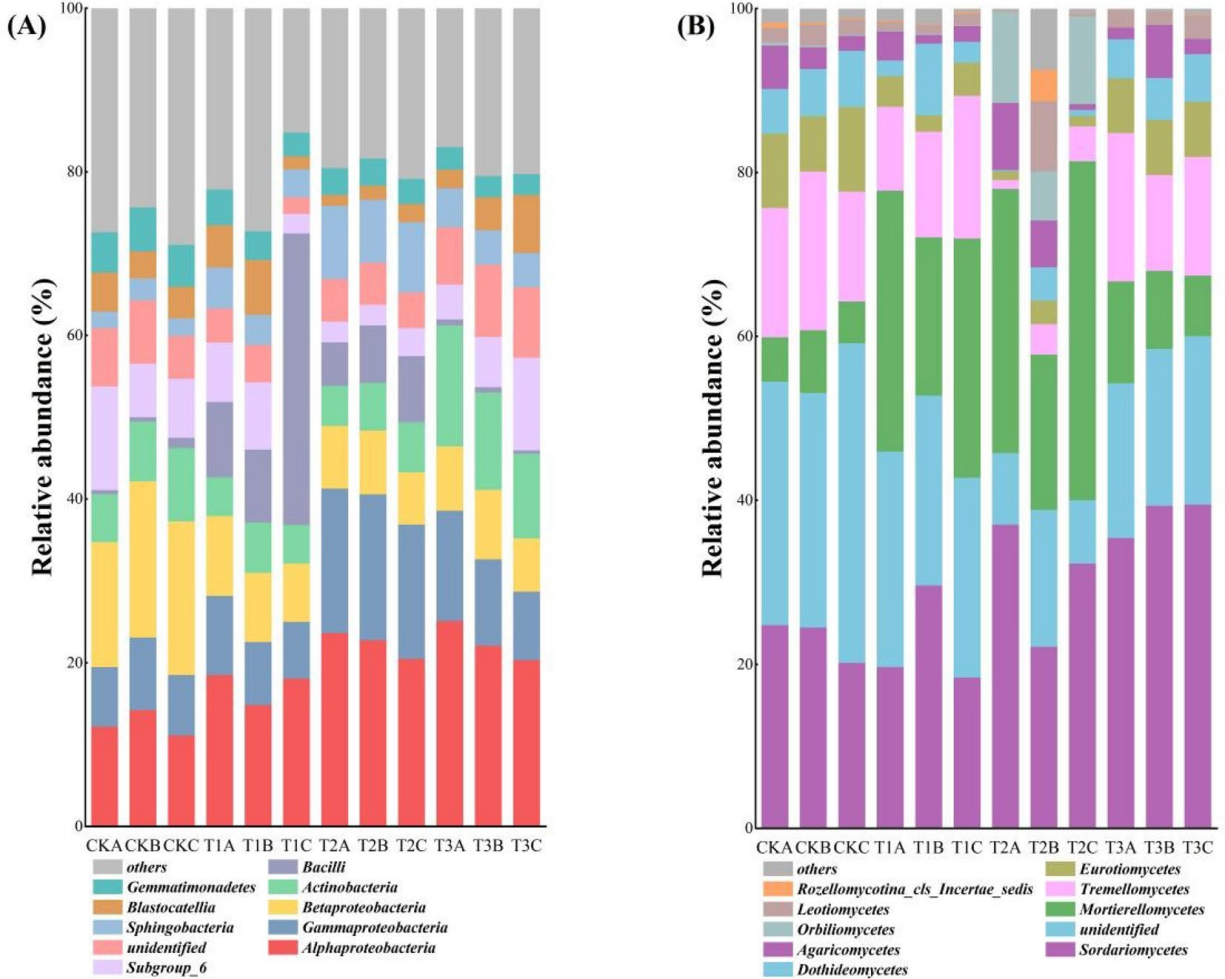
Bacterial **(A)** and fungal **(B)** classes community composition in different treated soils.

Among the bacterial communities, *Alphaproteobacteria* (12.52–22.53%), Gammaproteobacteria (7.85–17.28%), *Betaproteobacteria* (7.28–17.71%), *Actinobacteria* (5.18–12.34%), and *Bacilli* (0.60–17.90%) were the dominant classes. The relative abundance of *Alphaproteobacteria* and *Gammaproteobacteria* increased significantly while the relative abundance of *Betaproteobacteria* decreased significantly after JF-1-straw co-remediation (Fig. 4A) (P < 0.05). Likewise, *Sordariomycetes* (22.57–38.09%), *Mortierellomycetes* (6.06–30.84%), *Tremellomycetes* (3.02–16.17%), *Eurotiomycetes* (1.73–8.72%), and *Dothideomycetes* (1.66%-6.03%) were the dominant fungal phyla. The relative abundance of *Tremellomycetes*, *Eurotiomycetes*, and *Dothideomycetes* in the straw alone treatment was significantly higher compared with the T2 treatment (P < 0.05) but did not show a significant difference from the original soil relative abundance (Fig. 4B).

**Figure 5:**
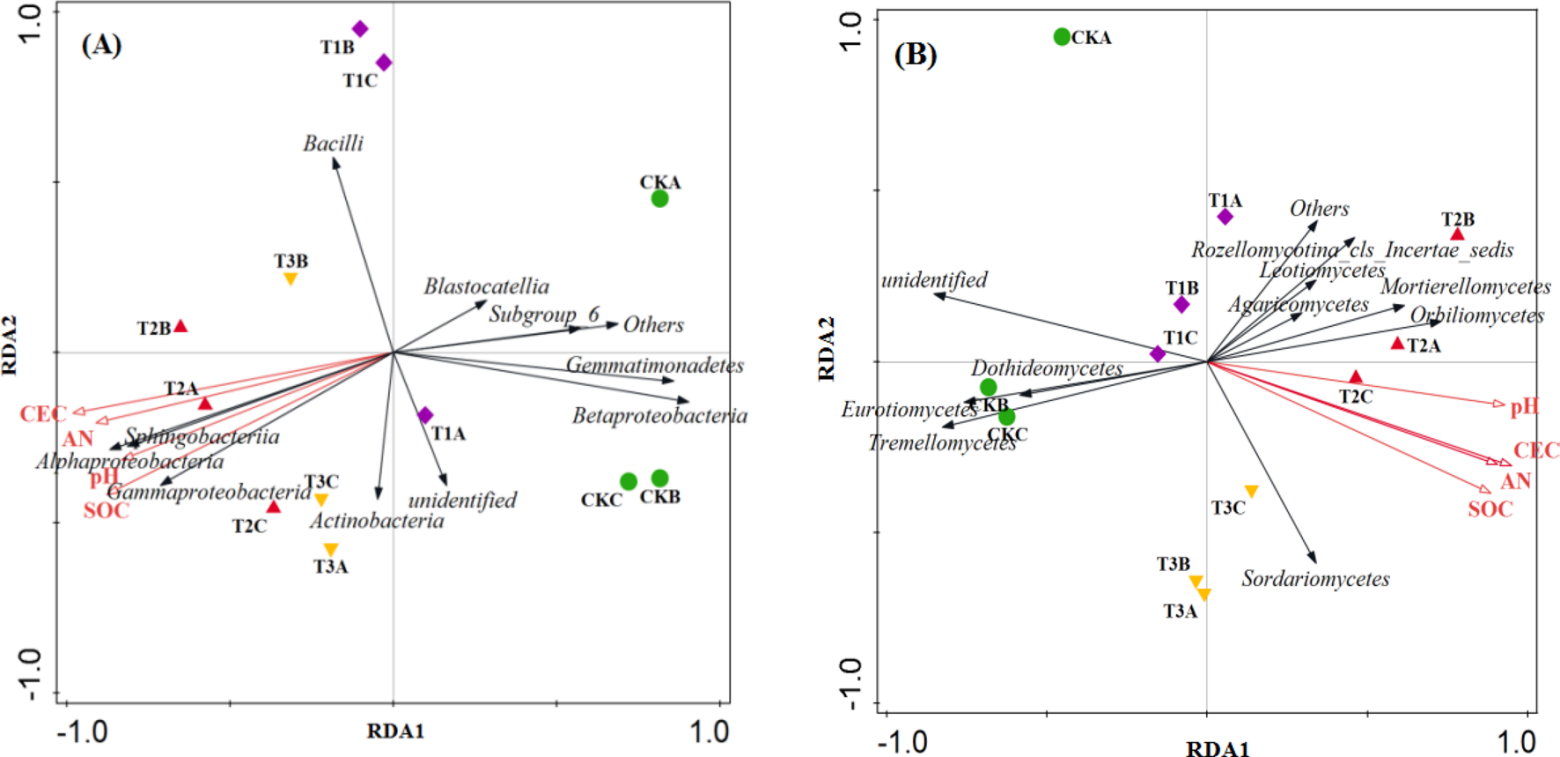
Redundancy analysis based on bacterial community **(A) **and fungal community **(B)** and environmental factors including the pH value, cation exchange capacity (CEC), soil organic carbon (SOC) and alkali-hydrolysable nitrogen (AN).

The relationship between the soil micro biota at the class level and four soil physicochemical factors, including pH value, cation exchange capacity (CEC), soil organic carbon (SOC) and alkali-hydrolysable nitrogen were shown in Fig. 5A,B, respectively. The change of soil cation exchange capacity was the primary driver underlying differences in microbial community structure (P < 0.05). For bacterial biota, *Alphaproteobacteria*, *Gammaproteobacteria* and *Sphingobacteria* were strongly positively correlated with pH value, cation exchange capacity, soil organic carbon and alkali-hydrolysable nitrogen (Fig. 5A). As for fungal biota, *Orbiliomycetes* was strongly positively correlated with pH value (Fig. 5B).

### Effect of different treatments on core soil microorganisms

**Figure 6:**
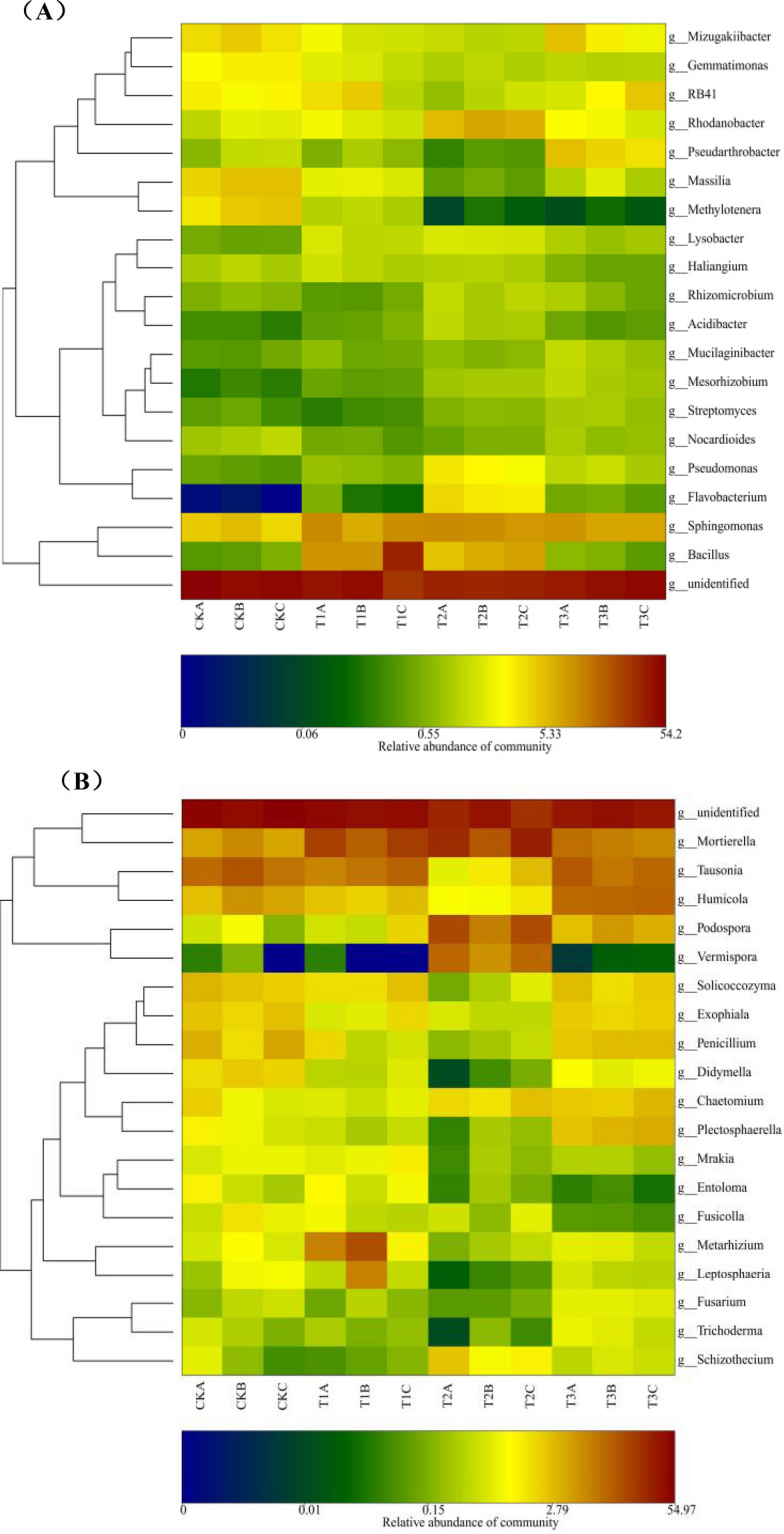
Effect of different treatments on core bacterial **(A)** and fungal **(B)** biota at genus level.

A total of 375 bacterial and 414 fungal genera were detected in the 12 soil samples, and the top 20 core bacterial and fungal genera were selected for the microbial community heat map to show the changes in their relative abundance under different remediation conditions (Fig. 6A,B). The remaining 355 bacterial and 394 fungal genera were grouped in the "other" group. *Massilia*, *Sphingomonas*, and *Methylotenera* were the dominant bacterial biota in the black soils of Northeast China, with 4978 (5.06%), 4780 (4.86%), and 4423 (4.50%) effective reads, respectively. Concerning fungi, *Tausonia*, *Mortierella*, and *Humicol*a were the dominant biota with effective reads of 7423 (11.39%), 3274 (5.02%), and 2811 (4.31%), respectively. Among these, the relative abundance of *Sphingomonas* and *Mortierella* significantly increased in the restored soil samples and more significantly in the soil co-remediated with the JF-1-straw combination, the effective reads were 9375 and 1647, accounting for 25.28 and 9.53% of the total number of soil bacteria and fungi, respectively (P < 0.05). *Bacillus*, which accounted for 0.44% of the total bacterial count in the original soil sample, was detected with 428 effective reads. After JF-1 remediation alone, and co-remediation with straw and JF-1 significantly increased the relative abundance of *Bacillus* with 17,524 and 6607 effective reads and 17.82 and 6.72% of the total bacterial count in the soil, respectively, but no statistically significant difference was found between the two treatments. Correspondingly, the relative abundance of the phytopathogenic fungi *Fusarium* was significantly (P < 0.05) suppressed after JF-1 alone or JF-1-straw co-remediation. It is noteworthy that the relative abundance of *Chaetomium* (fungi) increased significantly in the soil samples restored with straw alone, while *Plectosphaerella* (fungi) and *Fusarium* (fungi) also showed significant enrichment (P < 0.05), which was not observed in the other treatments. Correspondingly, *Pseudomonas* (bacteria), *Rhodanobacter* (bacteria), *Flavobacterium* (bacteria), *Schizothecium* (fungi), *Vermispora* (fungi) and *Podospora* (fungi) showed significant enrichment only in soil samples co-remediated with straw and JF-1(P < 0.05), while the relative abundance of *Lysobacter* (bacteria), *Rhizomicrobium* (bacteria) and *Chaetomium* (fungi) also showed some increase. In addition, the results also revealed that a number of bacterial and fungal genera showed different degrees of variation (Fig. 6A,B).

### The microbial functional diversity under maize straw and JF-1 applications

**Figure 7:**
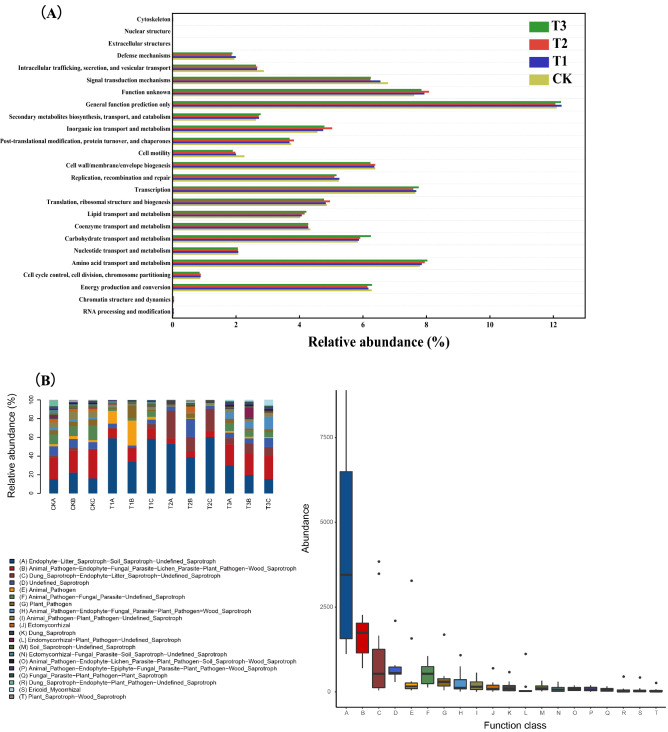
The bacterial functional diversity **(A)** and fungal functional diversity **(B)** under JF-1 and maize straw applications.

According to bacterial COG functional analysis (Fig. 7A), the abundance of lipid transport and metabolism significantly increased in T2 treatment and T3 treatment by 3.48% and 4.98% (P < 0.05), compared with CK treatment, but the abundance of cell cycle control/cell division/chromosome partitioning was decreased by 1.96% in T3 treatment (P < 0.05). Similarly, in T3 treatment, the translation, the abundance of ribosomal structure and biogenesis was decreased by 1.85%, while the abundance was significantly increased by 2.02% in T2 treatment (P < 0.05). Moreover, the straw-JF-1 combination increased some functional abundance of soil bacteria, among them the inorganic ion transport and metabolism, post-translational modification/protein turnover/chaperones and function unknown had the highest impact (P < 0.05). As for fungal community, the changes in the top twenty function of abundance were analyzed (Fig. 7B). Compared to other treatments, the straw-JF-1 combination showed the most effective inhibition of fungi associated with plant and animal pathogens.

### Effect of different treatments on disease resistance of maize at the seedling stage

**Figure 8:**
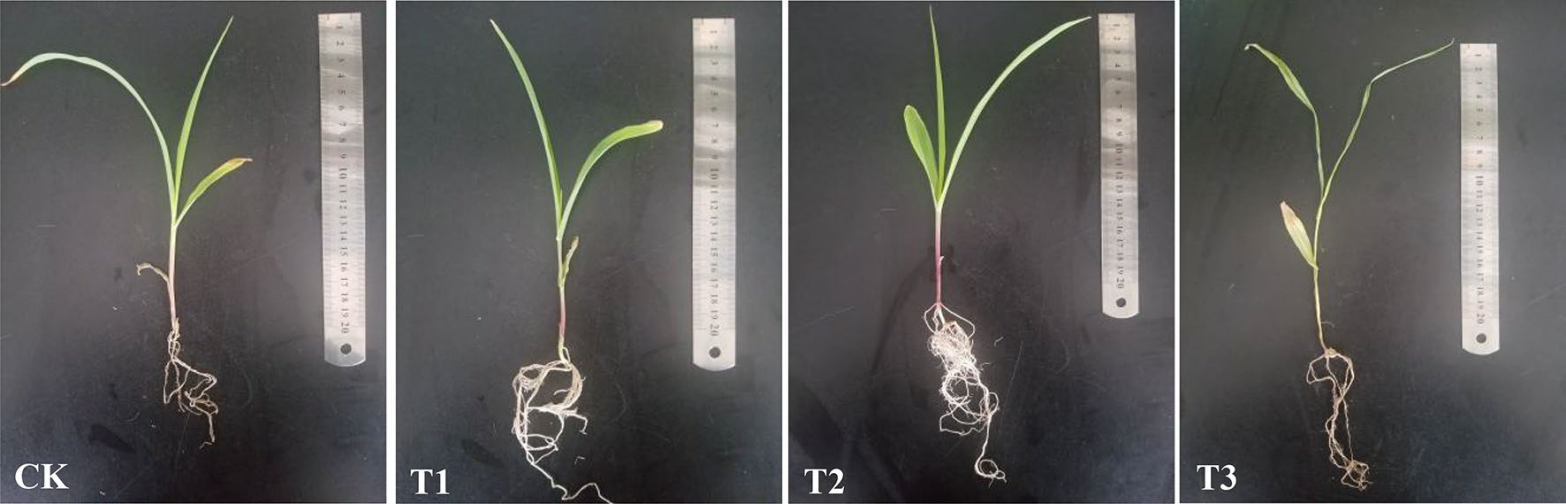
Effect of different treatments on disease resistance of maize at seedling stage.

Compared to CK, plant height, stem thickness, fresh weight, and dry weight showed a significant increase in T1 (by 8.83, 37.50, 15.66, and 63.63%, respectively) and T2 (by 12.85, 58.33, 23.23, and 72.72%, respectively) treatments (P < 0.05; Table 3). In addition, the T2 treatment showed a significant increase in stem thickness and fresh weight compared to the T1 treatment, by 15.15 and 6.55% respectively (P < 0.05). Compared to the CK treatment, the T3 treatment showed no significant difference in plant height, stem thickness, and dry weight, while fresh weight was significantly reduced by 13.64% (P < 0.05). The root systems of the CK and T3 treatments were affected to some extent, showing yellowing, shrinkage, poor development, and stunting with short lateral roots; the roots of the T3 treatment were brown and withered; the roots of the T1 and T2 treatments were healthier with white to cream color, and the T2 treatment had most developed root structure (Fig. 8).

**Table 3:** Effect of different treatments on disease resistance of maize at seedling stage.

Treatments	Plant height (cm)	Stalk thick (cm)	Fresh weight (g)	Dry weight (g)
CK	21.40 ± 0.80 b	0.24 ± 0.02 c	1.98 ± 0.14 c	0.11 ± 0.02 b
T1	23.29 ± 1.33 a	0.33 ± 0.02 b	2.29 ± 0.08 b	0.18 ± 0.03 a
T2	24.15 ± 0.75 a	0.38 ± 0.03 a	2.44 ± 0.08 a	0.19 ± 0.03 a
T3	21.35 ± 0.79 b	0.23 ± 0.02 c	1.71 ± 0.11 d	0.11 ± 0.03 b

Different letters indicate significant differences at p < 0.05 (according to Duncan’s multiple range test).

## Discussion

Enhancing plant disease and stress resistance by ensuring adequate nutrients is a common tool for sustainable farm management^24^. The assisted effect of JF-1 was more significant (P < 0.05) for pH enhancement than straw alone (Table 1). This could be due to the depletion of H^+^ and the release of metal ions that neutralized soil acidity during the mineralization of alkaline material in maize stalk^25^, the trend is consistent with the change in SOM content. Correspondingly, according to the analysis of bacterial function (Fig. 7A), it was found that straw alone and the combined application of straw and JF-1 significantly increased the relative abundance of soil bacterial inorganic ion transport and metabolism (P < 0.05). The direct return of straw to the soil is known to improve the levels of CEC, AN, AP, and AK. We found that the addition of *Bacillus amyloliquefaciens* JF-1 further increased that change (P < 0.05).

Studies have shown that straw return to the field can promote soil aggregation^26^. However, we found different results probably due to the slow degradation of straw in the low and arid climate of Northeast China, which resulted in low amounts of cohesive substances and polysaccharides that are important drivers of soil aggregation^27^. The synergistic effect of straw with JF-1 significantly enhanced the soil aggregation capacity (Fig. 1A). In addition, the SOM content of all sizes of aggregates was significantly enhanced in straw-added soils (Fig. 1B). This can be attributed to microbial mineralization of nutrients from the straw as suggested by Lou et al^28^.

Humic substances can alter the physicochemical and biological conditions of the soil^16^. Humic substances concentration was found positively correlated with the suppression of pathogenic fungi^29^. This study found that the straw-JF-1 combination significantly increased the soil levels of different humic substances compared to the direct return of straw-alone treatment (P < 0.05), as shown in Fig. 2. It has been reported that the addition of microbial agents to the composting process can effectively improve cellulosic organic substrate conversion into humus^30^. Therefore, the addition of JF-1 could have played a similar role, which is evident with an increase in the PQ values. Additionally, the highest structural simplification of HA and FA molecules in the soil humic substances was observed under the JF-1-straw combination treatment (P < 0.05). Notably, the higher content of carboxyl groups in the small molecules of humic substances is positively correlated with the fungal inhibitory ability of HA^16^. Also, the simplification of the HA molecular structure contributed to the increase in macro-aggregate (> 0.25 mm)^31^. Therefore, the addition of JF-1 to the straw return process was more effective in improving the decay of organic matter and the structure and the total amount of soil humic substances.

In this study, JF-1 or straw alone significantly increased the richness of the bacterial community, but did not affect the fungal community (Table 2). These results are consistent with De Vries et al^32^ that soil fungi are more stable and less affected by environmental changes compared to bacteria. Importantly, this study did not find the synergistic effect on the diversity and richness of the microbial community. Similarly, Sanaullah et al^33^ showed that microbial richness and diversity were not critical factors in the degradation of residual crops. Therefore, we inferred that the synergistic effect of the two accelerated the succession of soil microbial communities, while the separate applications of straw or JF-1 stimulated the microbial community only in the early stages. This is consistent with the results of PCoA that the synergistic effect of the straw-JF-1 combination on the soil microbiota is greater than the individual treatments (Fig. 3E,F). Bacteria such as *Alphaproteobacteria* and *Gammaproteobacteria* play a key role in the degradation of residues from maize, wheat, and alfalfa^34^. Also, the biocontrol potential of some of the substrates isolated from the *Gammaproteobacteria* class is of great interest^35^. *Betaproteobacteria* are less abundant in the soil of healthy plants than in the inter-root soil of diseased plants^36^. Fungi such as *Tremellomycetes*, *Eurotiomycetes*, and *Dothideomycetes* are the main decomposers of maize straw after return to the field, and their abundance increases followed by a decrease to a stable level^37^. The distribution of bacterial classes associated with degradation and biocontrol was more evenly balanced in the soil samples co-remediated with the straw-JF-1 combination (Fig. 4A,B). The composition of the fungal classes suggests that JF-1 addition ensured the straw degradation to the terminal stage. In this study, the RDA results showed that the increase in soil cation exchange capacity promoted the growth of some beneficial microorganisms, such as *Alphaproteobacteria*, *Gammaproteobacteria* and *Sphingobacteria* (Fig. 5A,B). Some metal ions in the soil participate in the life activities of microorganisms and play a key role in the expression of certain proteins and genes^38^.

As revealed in Fig. 6A,B, this study showed that the abundance of *Chaetomium*, which produces a variety of cellulose-degrading enzymes^39^, was significantly increased after the straw alone treatment. However, the abundance of plant pathogenic fungi *Fusarium* and *Plectosphaerella* also increased in the proportion of fungi biota. Similar to Fusarium, *Plectosphaerella* is also a genus of plant pathogenic fungi^40^. Therefore, the direct return of straw to the soil increased the risk of crop fungal diseases. This phenomenon can be attributed to the high C/N ratio of maize straw, which provides a large amount of organic carbon and nutrient substrates facilitating the multiplication of some saprophytic phytopathogenic fungi. Similar findings were reported by Voriskova and Baldrian^41^ in their study on leaf litter decomposition. Also, the straw may originally carry some pathogenic fungi to soil^42^. The significant increase (P < 0.05) in the relative abundance of *Bacillus* following JF-1 soil restoration may be attributed to JF-1 colonization in the soil; correspondingly, *Fusarium* was significantly inhibited (P < 0.05). The greatest increase was observed in the abundance of *Sphingomonas* and *Mortierella* post-straw-JF-1 treatment. The genus *Sphingomonas* is a widely reported biocontrol agent that colonizes plant roots and produces antifungal metabolites^43^. There is clear evidence that the genus *Sphingomonas* has a suppressive effect on *Fusarium*^44^. The genus *Mortierella* can directly inhibit the phytopathogenic fungi and was shown to suppress vanilla wilt by stimulating the growth of some functional antibiotic-producing microorganisms^45,46^. Importantly, this study found that the beneficial microbiota of *Pseudomonas*, *Rhodanobacter*, *Flavobacterium*, *Schizothecium*, *Vermispora*, and *Podospora* were significantly enriched only after straw-JF-1 combined soil treatment. The genus *Pseudomonas* can produce phenazine and 2,4-diacetylphloroglucinol, which can disrupt the physiological processes and metabolic activities of pathogenic microorganisms^47^. Also, *Rhodanobacter* is a potential genus of bacteria involved in disease suppression^48^. *Flavobacterium* can inhabit extreme environments and has good degradability of cellulose, hemicellulose, and lignocellulose^49^. Some species of the genus *Schizothecium* improved the growth of pumpkin under *Verticillium dahliae* stress^50^. Previous studies confirmed the antagonistic effect of *Vermispora* against *Meloidogyne graminicola*, *Rhizoctonia solani*, and nematodes^51^. Several species of the genus *Podospora* have antifungal functions^52^. Our results showed that the combined application of straw and JF-1 improved beneficial microbial biota, including *Lysobacter*, *Rhizomicrobium*, and *Chaetomium*. *Lysobacter* produces a variety of substances such as chitinase, glucanase, phenazines, and polycyclic tetramate macrolactam (PTM), which can control fungi and oomycetes induced plant diseases caused by^53^. *Rhizomicrobium* is beneficial for plant growth and facilitates the degradation of cellulose and aromatic hydrocarbons^54^. In conclusion, our research shows that the synergistic effect of JF-1 and straw effectively inhibits the growth of pathogenic fungi promoted by improving microbially relevant soil functions such as nutrient cycling, biometabolism of disease resistance factors, and degradation of organic matter.

The combined application of the bacterium JF-1 and straw significantly reduced the inhibitory effect of *Fusarium graminearum* on maize seedling growth (Table 3) (Fig. 8). Studies have shown that some species of the *Bacillus* genus produce antibiotics and fungal cell wall degrading enzymes such as chitinase and glucoamylase; while some species carry genes related to indole-3-acetic acid production and ferredoxin improving plant growth^55–58^. In addition, according to the analysis of bacterial functional analysis (Fig. 7A), the combined application of straw and JF-1 significantly increased the relative abundance of soil bacterial post-translational modification, protein turnover, chaperones and lipid transport and metabolism (P < 0.05). Studies have shown that some enzymatic proteins and lipopeptides act as bioinhibitors of plant pathogenic fungi, induce the development of plant defence mechanisms and promote plant growth^59–62^. According to FunGuild fungal function analysis (Fig. 7B), compared to other treatments, the combination of straw and JF-1 showed the most significant inhibition of fungi associated with plant and animal pathogens (P < 0.05). The suppression of soil-borne diseases consists mainly of conventional suppression (via soil quality and environmental microorganisms) and specific suppression (via particular microorganism) methods^15^. In conclusion, we found that the synergistic effect of maize straw and the bacterium JF-1 enhanced both the conventional and specific suppression of the soil and thereby reduced the negative effects of *Fusarium graminearum* and promoted the growth of maize seedlings.

## Conclusions

This paper reveals that the combined application of maize straw and the bacterium JF-1 can significantly improve soil quality, disease resistance, soil fertility, soil aggregation capacity, SOM content of soil aggregates, quality of humic substances, and beneficial changes in soil microbial communities (e.g. *Sphingomonas*, *Mortierella*, *Pseudomonas*, *Rhodanobacter*, *Flavobacterium*, *Vermispora*, etc.), while the relative abundance of disease-causing fungi (*Fusarium* and *Plectosphaerella*) was significantly reduced (P < 0.05). The synergistic effect of maize straw and JF-1 was also found effective against *Fusarium graminearum* induced damage to maize seedling in pot trials. In conclusion, this study provides an effective and low-cost soil remediation method for the mitigation of maize diseases.

## Methods

### Field trial

The study was conducted at the experimental site of Jilin Agricultural University (125°23′39″E and 43°48′39″N) in a temperate continental humid climate. The soil conditions were as follows: soil type, mollisol; pH, 6.05; available nitrogen (AN), 83.17 mg·kg^−1^; available phosphorus (AP), 31.70 mg·kg^−1^; available potassium (AK), 143.68 mg·kg^−1^; and soil organic carbon (SOC), 11.36 g·kg^−1^. The field experiment started on 1 October 2019 and was carried out in four plots of 4 m^2^ each. There were four treatments: (1) T1 treatment: JF-1 microbicide alone; (2) T2 treatment: straw and JF-1 microbicide together; (3) T3 treatment: straw alone; (4) CK treatment: blank control. For each treatment, the soil was placed in nylon bags (30 cm diameter × 15 cm height) and buried in the tillage layer of each plot, with three replicates. To inoculate with JF-1 microbial suspension, 100 mL of 1 × 10^8^ CFU·mL^−1^ of bacterial solution was sprayed evenly on the soil surface of each plot for the T1 and T2 treatments, respectively, while CK and T3 were performed with 100 mL of sterile water, respectively. For the T2 and T3 treatments, the soil in the nylon bags was evenly mixed with maize straw crushed to 3–5 cm (0.70% addition rate). *Bacillus amyloliquefaciens* JF-1 (GenBank login number: MW578378) was provided by the Agricultural Pollution Prevention and Control Research Unit, School of Resources and Environment, Jilin Agricultural University. The maize stalk addition rate was 12 t hm^-2^ of the field returned straw. The soil, collected in nylon bags, was used to determine soil fertility, agglomerates, and humic substances in pot trials, and stored at-80 °C for the extraction of soil microbial DNA.

### Determination of soil physicochemical properties

Soil pH was determined using a pH meter (PHS-3E, Shanghai Precision Scientific Instruments Co., Ltd.) using a 1:2.5 soil–water mixture. The SOC content was determined by the K_2_Cr_2_O_7_–H_2_SO_4_ heating method. The soil contents of AN, AP, AK and CEC were determined as described previously^63,64^. The soil aggregates were classified into large macro-aggregates (> 2 mm), small macro-aggregates (2–0.25 mm), micro-aggregates (0.25–0.053 mm) and silt/clay fraction (< 0.053 mm) following the Cambardella and Elliott's wet sieving method^65^. Humic substances (humic acid (HA), fulvic acid (FA), and humin (HM)) were extracted according to the method of Zhang et al^66^. The ΔlgK values of HA and FA were computed as the log difference of absorbance at 400 and 600 nm; the PQ value was computed as PQ = HA-C/(HA-C + FA-C) × 100.

### Microbial DNA extraction and Illumina MiSeq sequencing

DNA was extracted from the soil with a PowerSoil DNA Isolation Kit (MoBio Laboratories, Carlsbad, CA, USA). DNA concentrations and purities were quantified by NanoDrop 2000 UV–Vis spectrophotometer (Thermo Fisher Scientific, Wilmington, USA). PCR amplification was performed using the bacterial V3-V4 region-specific primers (338F 5'-ACTCCTACGGGAGCAG-3'; 806R 5'-GGACTACHVGGGTWTCTAAT-3') and fungal ITS specific primers (ITS1F 5'-CTTGGTCATTAGAGGAAGTAA-3'; ITS2 5'-TGCGTTCTTCATCGATGC-3'). PCR amplification conditions were as follows: (1) 50 μL PCR amplification reaction contained 15 μL 2 × Taq Master Mix, 1 μL of 10 μM Bar-primer F, 1 μL of 10 μM Bar-primer R, and 10–20 ng genomic DNA template filled with water; (2) Illumina bridge PCR compatible primers were introduced in the 50 μL amplification system, and the reaction contained 15 μL 2 × Taq Master Mix, 1 μL of 10 μM primer F, 1 μL of 10 μM primer R, and 20 ng PCR product generated from the first step and rest of the volume was completed with distilled water. The PCR products were analyzed on 1% agarose gels. The sequencing library was constructed based on the manufacturer’s instructions and sequencing was accomplished by the Beijing Auwigene Gene Technology Co. using an Illumina Miseq PE250 platform.

### Processing of sequencing results

The raw data were first screened to remove sequences < 200 bp in length and chimeras to obtain good quality sequences as clean-tags. Program vsearch 2.7.1 was used to perform OTU (Operational Taxonomic Units) classification on the processed sequences, and OTUs at 97% similarity level were clustered. Bioinformatic analysis was performed based on the results of OTUs clustering analysis^67^; rarefaction analysis was performed using mothur software package v.1.30.1^68^. Rarefaction curves were plotted using R, and alpha diversity analysis was performed. Venn diagrams were plotted by R version 3.6.1 to count the number of common and unique OTUs to multiple samples. To analyze the differences and similarities between samples, PCoA (principal coordinate analysis) was performed based on the Bray–Curtis method. Histograms of the relative abundance of the top 10 OTUs at the class level were plotted using Origin 2019. To compare the composition and variability of species between treatments at the genus levels, distance calculations and cluster analysis were carried out using the vegdist and hclust programs, and the heat map was plotted with the top 20 OTUs using the vegan package of R version 3.6.1. redundancy analysis (RDA) was performed using CANOCO 5 software^69^. Bacterial function analysis was carried out with COG functional classification. FUNGuild v1.0 was used to determine the functional groups of fungi and the top twenty taxa of relative abundance were selected for analysis.

### Pot trials

Pot trials were carried out at the end of the field trial on 1 May 2020. Soil samples were first collected from the nylon bags of the different treatments using the quadrat method. The soil samples were placed in pots of 10 cm diameter and 15 cm height and divided into four treatments, each with three replicates. The soil samples were inoculated with a highly pathogenic *Fusarium graminearum* spores suspension with a concentration of 1 × 10^8^ CFU·mL^−1^ at a ratio of 1:50 (spore suspension: soil, V/m). Maize seeds (Kennian NO. 1) were procured from Beijing Jinnong Fengyuan Seeds Co., Ltd. (seeds and plants were used for only research purpose with proper permission). Three maize seeds were placed in each pot and the height, stem thickness, fresh weight, and dry weight of maize seedlings were measured after 15 days of incubation.

### Statistical analysis

Data were plotted with Origin 2019 (OriginLab, USA) and are shown mean values. Microsoft Excel 2010 was used for basic data processing. SPSS 18.0 (SPSS Inc, Chicago, Illinois) software was used for data analysis and processing. One-way ANOVA LSD (the least significant differences) was used for the analysis of significant differences at the P < 0.05.

### Complies with international, national and/or institutional guidelines

Experimental research and pot studies on soil and plants (seeds and plants were used for only research purpose with proper permission), comply with relevant institutional, national, and international guidelines and legislation.
